# Neuroprotective Effects of Cerium Oxide Nanoparticles During Spaceflight

**DOI:** 10.1002/smsc.70271

**Published:** 2026-03-31

**Authors:** Alessio Carmignani, Attilio Marino, Matteo Battaglini, Nicoletta Di Leo, Elisa Carrubba, Michele Balsamo, Giovanni Valentini, Gabriele Mascetti, Serena Perilli, Francesco De Boni, Sergio Marras, Mirko Prato, Giada Graziana Genchi, Gianni Ciofani

**Affiliations:** ^1^ Smart Bio‐Interfaces Istituto Italiano di Tecnologia Pontedera Italy; ^2^ Kayser Italia S.r.l. Livorno Italy; ^3^ Agenzia Spaziale Italiana Roma Italy; ^4^ Materials Characterization Facility Istituto Italiano di Tecnologia Genova Italy; ^5^ Department of Bioscience, Biotechnology and Environment University of Bari “Aldo Moro” Bari Italy

**Keywords:** microgravity, nanoceria, neuroprotection, oxidative stress, spaceflight

## Abstract

The spaceflight environment exposes biological systems to microgravity and cosmic radiation, which are factors known to induce oxidative stress and neurodegenerative processes. As the central nervous system is highly susceptible to disruptions in redox homeostasis, the development of effective strategies to safeguard astronaut health and cognitive function during extended space missions has become imperative. In this study, we investigate cerium oxide nanoparticles (nanoceria, NC) as an antioxidant agent for human neuron‐like cells aboard the International Space Station. Nanoceria demonstrated excellent biocompatibility, strong antioxidant properties, and the ability to stimulate neurite extension under both Earth gravity and simulated microgravity. Following the return of the samples to Earth, transcriptomic analyses revealed that nanoceria effectively counteracted the detrimental transcriptional alterations triggered by spaceflight stressors, thereby maintaining neuronal homeostasis. Importantly, the expression of genes involved in antioxidant defense, mitochondrial activity, and dopamine metabolism remained stable in nanoceria‐treated neurons, in contrast to the dysregulation observed in untreated controls. These findings position cerium oxide nanoparticles as promising antioxidant neuroprotectants for long‐duration space missions and related neurodegenerative conditions.

## Graphical abstract

Cerium oxide nanoparticles are investigated as antioxidant neuroprotectants for neurons exposed to the spaceflight environment aboard the International Space Station. The treatment preserves neuronal morphology, supports neurite outgrowth, and stabilizes gene networks involved in antioxidant defense, mitochondrial function, and dopamine metabolism, revealing a nanotechnology‐based strategy to counteract oxidative stress and neuronal dysregulation induced by microgravity and cosmic radiation.

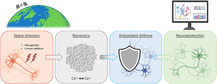

## 1 Introduction

Long‐duration spaceflight exposes astronauts to both microgravity (µ*g*) and cosmic radiation (CR), the main environmental stressors implicated in neurophysiological disturbances in low Earth orbit [1, 2]. Microgravity can disrupt cellular mechanotransduction and promote oxidative stress in neural tissues, contributing to neuroinflammatory responses and neuronal damage [3]. Concurrently, chronic exposure to CR exacerbates oxidative damage and can lead to DNA damage in neural cells [4]. Evidence from astronaut studies and model organisms also indicates that spaceflight conditions alter dopamine neurotransmission and gene expression in the brain, which may underlie motor coordination issues observed after flight [5]. These findings thus raise concerns that oxidative stress and related neurodegenerative processes during space missions could impair astronauts’ cognitive and motor function on a long term [6].

Despite the valuable information gained from simulated microgravity (sµ*g*) platforms and from radiation studies conducted on Earth, these approaches cannot fully capture the intricate physio‐/pathological alterations that occur during actual spaceflight. Indeed, transcriptomic and proteomic investigations in animal models have shown that the molecular responses triggered by real µ*g* often significantly diverge from those induced under simulated µ*g* [7]. Consequently, a considerable knowledge gap persists in differentiating the unique effects of real µ*g* from those produced by sµ*g* and CR on the central nervous system (CNS), emphasizing the critical need to corroborate ground‐based findings with in‐flight experimental data.

In this context, neuron‐like cells can serve as a well‐established in vitro model for dopaminergic neurons in Parkinson's disease research also due to their sensitivity to oxidative stress [8]. Simulated µ*g* experiments have shown that gravitational unloading can increase intracellular reactive oxygen species (ROS) levels and trigger cellular dysfunction. For instance, Qu et al. demonstrated that under sµ*g* conditions, SH‐SY5Y cells experience an increment in oxidative stress, which could be mitigated by antioxidant treatments [9]. Specifically, certain flavonoids (*e.g.*, isorhamnetin, luteolin) prevented µ*g*‐induced ROS accumulation and subsequent neuron‐like cell damage, underscoring the role of oxidative stress in µ*g*‐induced neurodegeneration, and suggesting that antioxidant‐based interventions can be effective against gravitational unloading effects in vitro [9].

Nanozymes have emerged as a novel class of countermeasures against oxidative stress due to their ability to directly scavenge free radicals or modulate cellular antioxidant responses [10]. In particular, cerium oxide nanoparticles (nanoceria, NC) can cycle between Ce^3+^ and Ce^4+^ oxidation states on their surface, enabling them to repeatedly scavenge superoxide and peroxyl radicals, and other ROS [11]. This regenerative redox activity endows nanoceria with prolonged antioxidant effects in biological environments, unlike conventional antioxidants that are consumed stoichiometrically [12]. Moreover, nanoceria have already shown neuroprotective potential in various models of oxidative stress and injury, protecting neuronal cells in a traumatic brain injury model, and reducing cell death and calcium dysregulation by preserving the endogenous antioxidant system [13]. In the PC12 neuronal cell model, for instance, nanoceria have been reported to support cell health, by increasing dopamine secretion in proliferative cells and by promoting neurite outgrowth and maturation during cell differentiation [14]. These properties make nanoceria an intriguing candidate for counteracting µ*g*/radiation‐induced oxidative stress, potentially offering long‐lasting ROS scavenging and support for mitochondrial activity during spaceflight [15, 16].

Here, we present a comprehensive space biology study testing the neuroprotective efficacy of nanoceria on differentiated SH‐SY5Y cells under real spaceflight conditions. The PROMETEO 2 experiment was conducted aboard the International Space Station (ISS), by using the same hardware and experimental design of the prior PROMETEO experiment [17], to allow a direct comparison between two nanotechnological antioxidants. Cells were exposed to µ*g* and CR on the ISS, and the same spaceflight timeline was then applied to ground‐based controls in sµ*g* and Earth gravity (1 g) conditions, thus allowing the of the potential NC shielding effect against CR. Nanoceria were administered to test their ability to mitigate oxidative stress, cellular damage, and gene expression dysregulation induced by the space environment, showing the ability to preserve cell viability, reduce oxidative damage, and maintain neuronal morphology and gene expression profiles closer to those of Earth controls. Our results could have relevance beyond space medicine, offering insights not only for preserving astronaut health on prolonged missions, but also for advancing therapeutic strategies against CNS disorders driven by oxidative stress on Earth.

## 2 Results

### 2.1 Nanoparticle Characterization

**FIGURE 1:**
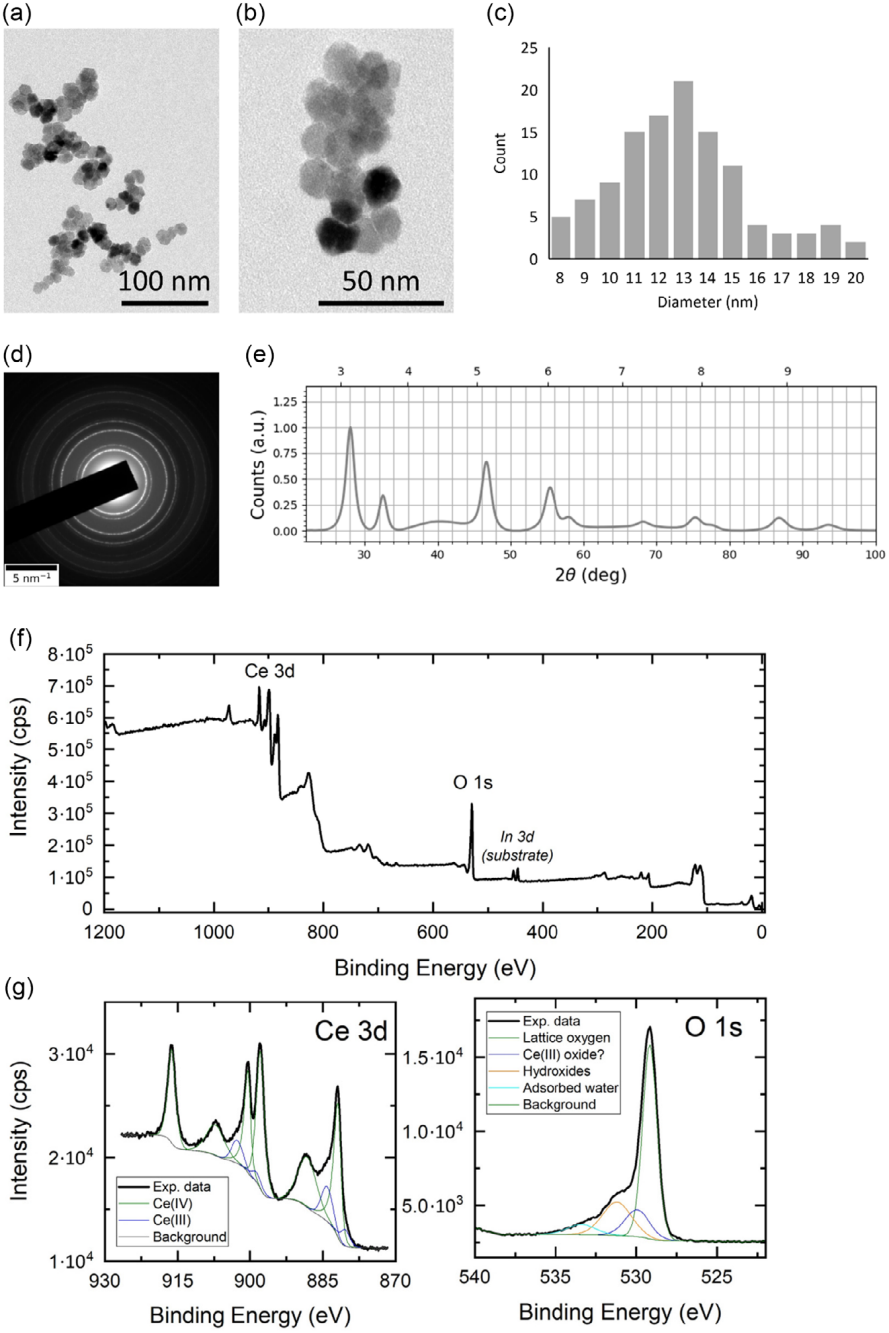
Nanoceria characterization. (a–b) Representative TEM images. (c) Size distribution derived from TEM images. (d) SAED pattern and (e) profile. (f) XPS representative survey spectrum and (g) XPS narrow spectrum. SAED = Selected area electron diffraction; TEM = transmission electron microscopy; XPS = X‐ray photoelectron spectroscopy.

Transmission electron microscopy (TEM; Figure 1b,c) showed that the synthesized nanoceria had an average diameter of 12.9 ± 2.8 nm (Figure 1c), corresponding to the consistent output of our synthesis protocol and therefore used as a single, well‐characterized formulation for the biological experiments in this study. The selected area electron diffraction (SAED) pattern was consistent with ICSD#24887, displaying the characteristic cerium oxide peaks at approximately 28°, 33°, 47°, 55°, 58°, 68°, 75°, 78°, 87°, and 94° (Figure 1d,e) [18, 19]. The wide‐scan X‐ray photoelectron spectroscopy (XPS) spectrum (Figure 1f) exhibited a Ce 3d signal at 883 eV and an O 1s signal at 530 eV, along with minor features attributable to residual unreacted precursors [20]. High‐resolution analysis of the Ce 3d region (Figure 1g) enabled estimation of the relative abundance of Ce^3+^ and Ce^4+^, the two redox states implicated in the antioxidant behavior of nanoceria in vitro, with the predominance of Ce^4+^ (16% of Ce^3+^ and 84% Ce^4+^) indicating catalase‐like activity in the synthesized nanoparticles [21, 22].

Upon NC coating with fetal bovine serum (FBS), dynamic light scattering (DLS) analysis showed a hydrodynamic diameter (*D*_h_) of 203.8 ± 2.4 nm and a polydispersity index (PDI) of 0.051 ± 0.029 (Figure S1a). The *ζ*‐potential resulted to be of −25.7 ± 0.4 mV (Figure S1b). To verify colloidal stability under biologically relevant conditions, nanoceria were dispersed in differentiation medium (composition detailed in the Experimental Section). Over the entire monitoring period, neither *D*_h_ nor PDI showed appreciable variation, indicating that the nanoparticles remain highly stable in a physiological‐like environment, thanks to the FBS coating (Figure S2).

### 2.2 Evaluation of Nanoparticle–Cell Interactions

To characterize how nanoceria interacts with neuron‐like cells, we first examined the impact on cell viability. Cultures were exposed to a range of nanoceria concentrations (0–200 µg/mL), and viability was assessed at 48 and 96 h by using a fluorometric assay that quantifies double‐stranded DNA content as an estimate for viable cell number (Figure S3a). Under standard gravity conditions, none of the tested concentrations produced a statistically significant decrement in cell viability (*p* > 0.05). The same set of treatments was then repeated under sµ*g* by using an RPM. In this condition, nanoceria exposure did not alter viability at 48 h (*p* > 0.05). Conversely, after 96 h, a significant reduction in viability emerged at the highest dose tested (200 µg/mL; *p* < 0.01, Figure S3b). Based on these results, 100 µg/mL was chosen as the working concentration for subsequent experiments.

To further validate the antioxidant behavior of cerium oxide nanostructures, we quantitatively evaluated their impact on intracellular reduced glutathione (GSH) levels. Under 1 g conditions (Figure S4a), control cells contained 5.95 ± 0.05 ng of GSH (quantity normalized *per* cell number), whereas cells exposed to nanoceria showed slightly elevated GSH levels (6.37 ± 0.20 ng). When a pro‐oxidant stressor (*tert*‐butyl hydroperoxide, TBH) was administered, GSH content dropped to 4.25 ± 0.15 ng in untreated cells, while nanoceria‐treated cells preserved significantly higher GSH levels of 5.44 ± 0.10 ng (*p* < 0.001). In sµ*g* condition (Figure S4b), baseline GSH amounted to 4.85 ± 0.13 ng in control cultures and 5.13 ± 0.40 ng in nanoceria‐exposed cells. Following oxidative stress induction, GSH levels decreased to 3.46 ± 0.08 ng in untreated cells, whereas nanoceria‐treated cells retained higher GSH content (4.22 ± 0.26 ng, *p* < 0.01).

### 2.3 Influence on Neuronal Differentiation and Neurite Outgrowth

**FIGURE 2:**
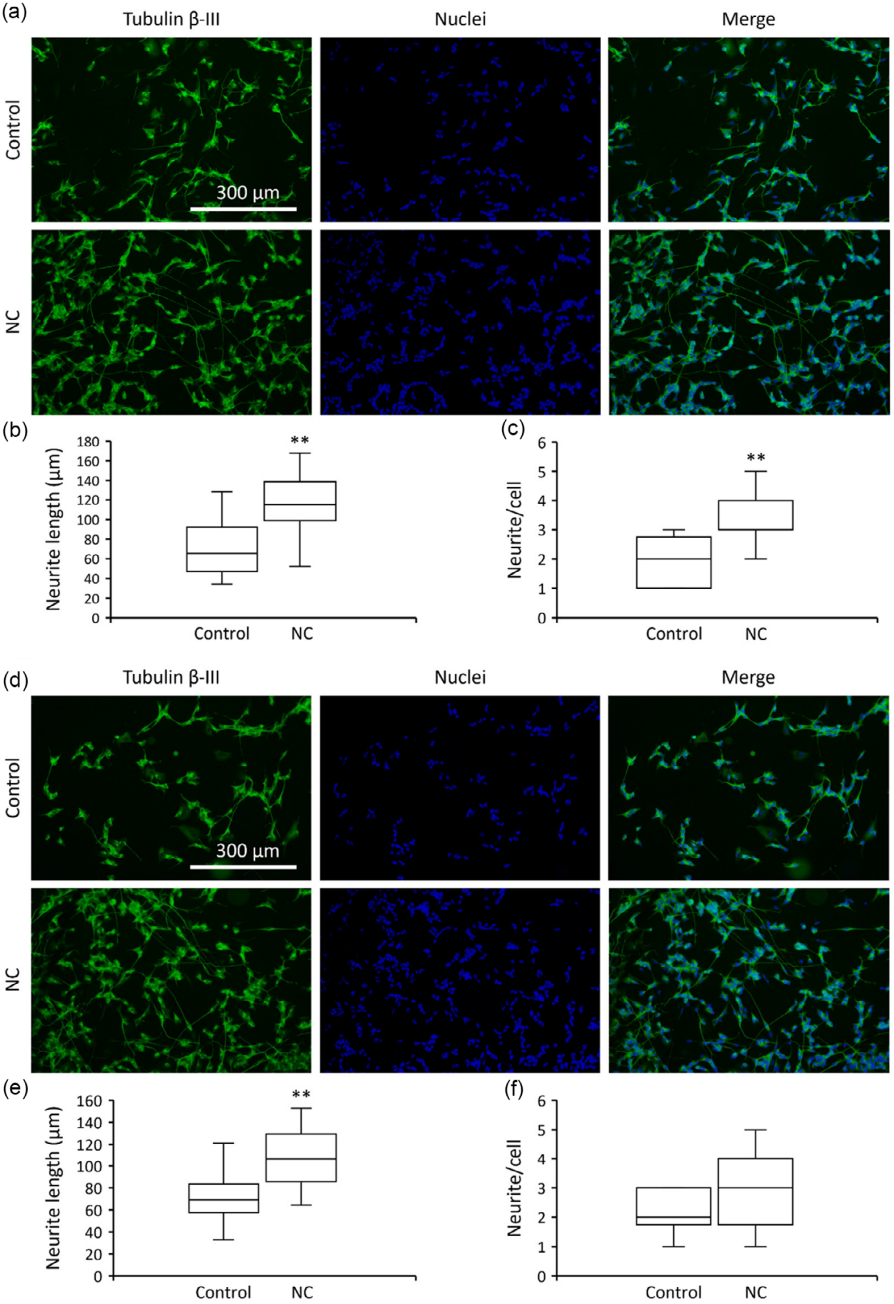
Analysis of nanoceria effect on neurite outgrowth. (a) Representative epifluorescence images (tubulin β‐III in green, nuclei in blue), (b) median neurite length, and (c) neurite/cell ratio for cultures in 1*g*. (d) Representative epifluorescence images (tubulin β‐III in green, nuclei in blue), (e) median neurite length, and (f) neurite/cell ratio for cultures in sµ*g* (** *p* < 0.01).

The ability of nanoceria to promote neuronal differentiation and neurite outgrowth was examined by epifluorescence microscopy (Figure 2a). After 96 h in differentiation medium, neuron‐like cells displayed a median neurite length of 65.7 ± 2.0 µm. Supplementation of the medium with 100 µg/mL nanoceria led to a marked increase in neurite length, reaching 115.0 ± 1.1 µm (*p* < 0.01; Figure 2b). In parallel, the median number of neurites *per* cell rose from 2.0 ± 0.1 in untreated cultures to 3.0 ± 0.1 in nanoceria‐treated cells (*p* < 0.01; Figure 2c). A comparable trend was observed under sµ*g* conditions (Figure 2d). In the absence of nanoparticles, cells exhibited a median neurite length of 69.1 ± 1.5 µm and 2.0 ± 0.1 neurites *per* cell (*p* < 0.01; Figure 2e). When nanoceria were added under the same conditions, both parameters increased, with neurite length reaching 106.2 ±  1.0 µm and the number of neurites *per* cell rising to 3.0 ± 0.1 (*p* > 0.05; Figure 2f).

### 2.4 In‐Flight Experiments

**FIGURE 3:**
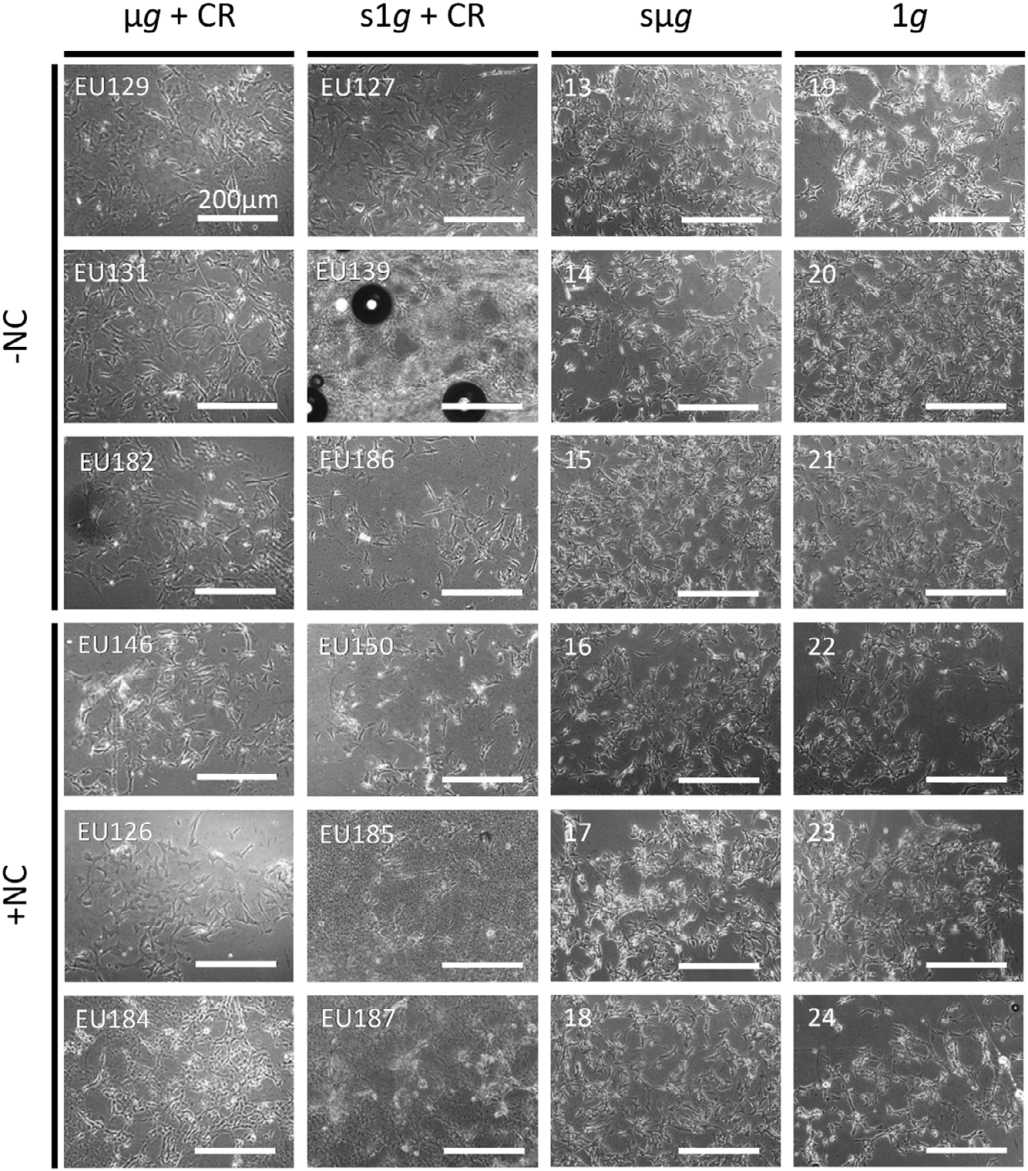
Representative phase contrast optical microscopy images of neuron‐like cells at the experiment endpoint (*i.e.*, after spaceflight, cold stowage, and following deintegration on ground). µ*g* = microgravity, CR = cosmic radiation, s1*g* = simulated Earth gravity, sµ*g* = simulated microgravity, 1*g* = Earth gravity, EU = experimental unit.

As shown in Figure S5, culture temperatures were consistently maintained at 37°C up to the insertion of the experiment containers (ECs), housing the experimental units (EUs) for automated in‐flight handling of the cell cultures, into the Kubik incubator aboard the ISS at 63 h after payload launch (L + 63 h, where “L” denotes the SpaceX Falcon 9 launch time). By the end of the experimental timeline, neuronal cultures remained viable and firmly adherent (Figure 3). The entire spaceflight protocol was reproduced on ground under sµ*g* conditions generated by 3D clinorotation on an RPM. Under these simulated conditions, cultures were found to be viable and adherent at the conclusion of the experiment, as well (Figure 3).

### 2.5 RNA Extraction and Next‐Generation Sequencing

After RNA extraction, purification, and quality control, we carried out transcriptomic profiling to determine which gene pathways were modulated by µ*g*, CR, and nanoceria treatment. The number of differentially expressed genes (DEGs) identified in each of the seven predefined pairwise comparisons among experimental conditions is shown in Table S1. In neuron‐like cells, exposure to µ*g* (A *vs.* C) and CR only (A *vs.* E) accounted for 978 and 3,492 DEGs, while the combined exposure to µ*g* and CR (A *vs.* G) yielded 3,084 DEGs. Nanoceria alone at 1 g (H *vs.* G) led to differential expression of 67 genes, whereas nanoceria treatment during spaceflight (B *vs.* A) resulted in 852 DEGs. Examination of nanoceria‐mediated rescue under µ*g* (B *vs.* C) and µ*g* + CR conditions (B *vs.* G) revealed 364 and 477 DEGs, respectively.

**FIGURE 4:**
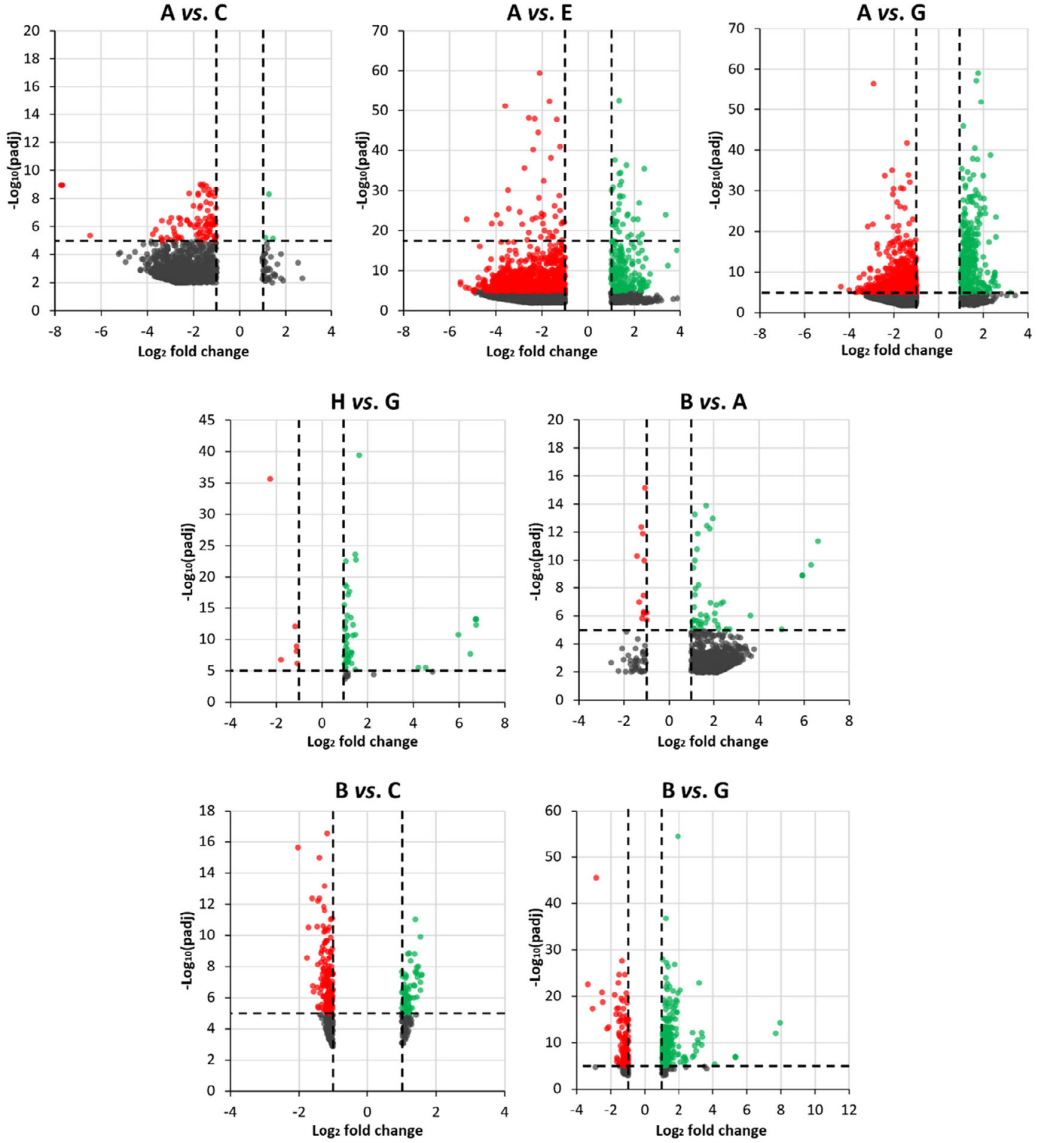
Scatter plots for the seven selected comparisons. Each volcano plot shows significance (*y*‐axis, as log_10_ of the adjusted *p*‐value, padj) and overexpression change (*x*‐axis, as log_2_ fold change) for all genes (each represented by a dot) in a given comparison. Significantly up‐ or downregulated genes are reported in green or red, respectively, while the remaining genes are indicated in black. Experimental classes: A (‐NC, µ*g*, +CR), B (+NC, µ*g*, +CR), C (‐NC, s1*g*, +CR), D (+NC, s1*g*, +CR), E (‐NC, sµ*g*, ‐CR), F (+NC, sµ*g*, ‐CR), G (‐NC, 1*g*, ‐CR), H (+NC, 1*g*, ‐CR).

**FIGURE 5:**
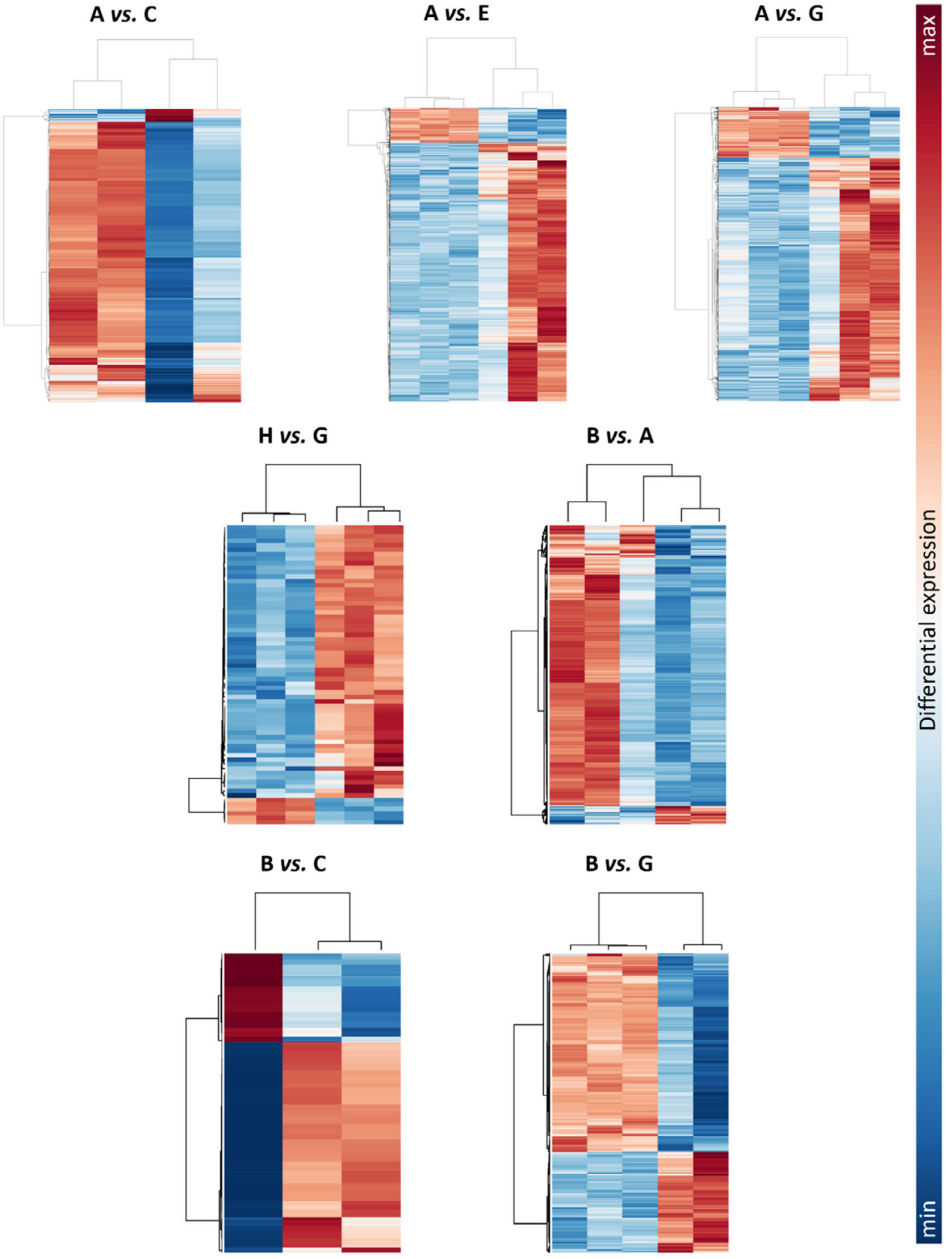
Heatmaps for the seven selected comparisons. Expression values for such genes are reported as colored bars. The hotter the color, the higher the fold change (FC; upregulated genes within each map are indicated in red, and downregulated genes are shown in blue). A (‐NC, µ*g*, +CR), B (+NC, µ*g*, +CR), C (‐NC, s1*g*, +CR), D (+NC, s1*g*, +CR), E (‐NC, sµ*g*, ‐CR), F (+NC, sµ*g*, ‐CR), G (‐NC, 1*g*, ‐CR), H (+NC, 1*g*, ‐CR).

**FIGURE 6:**
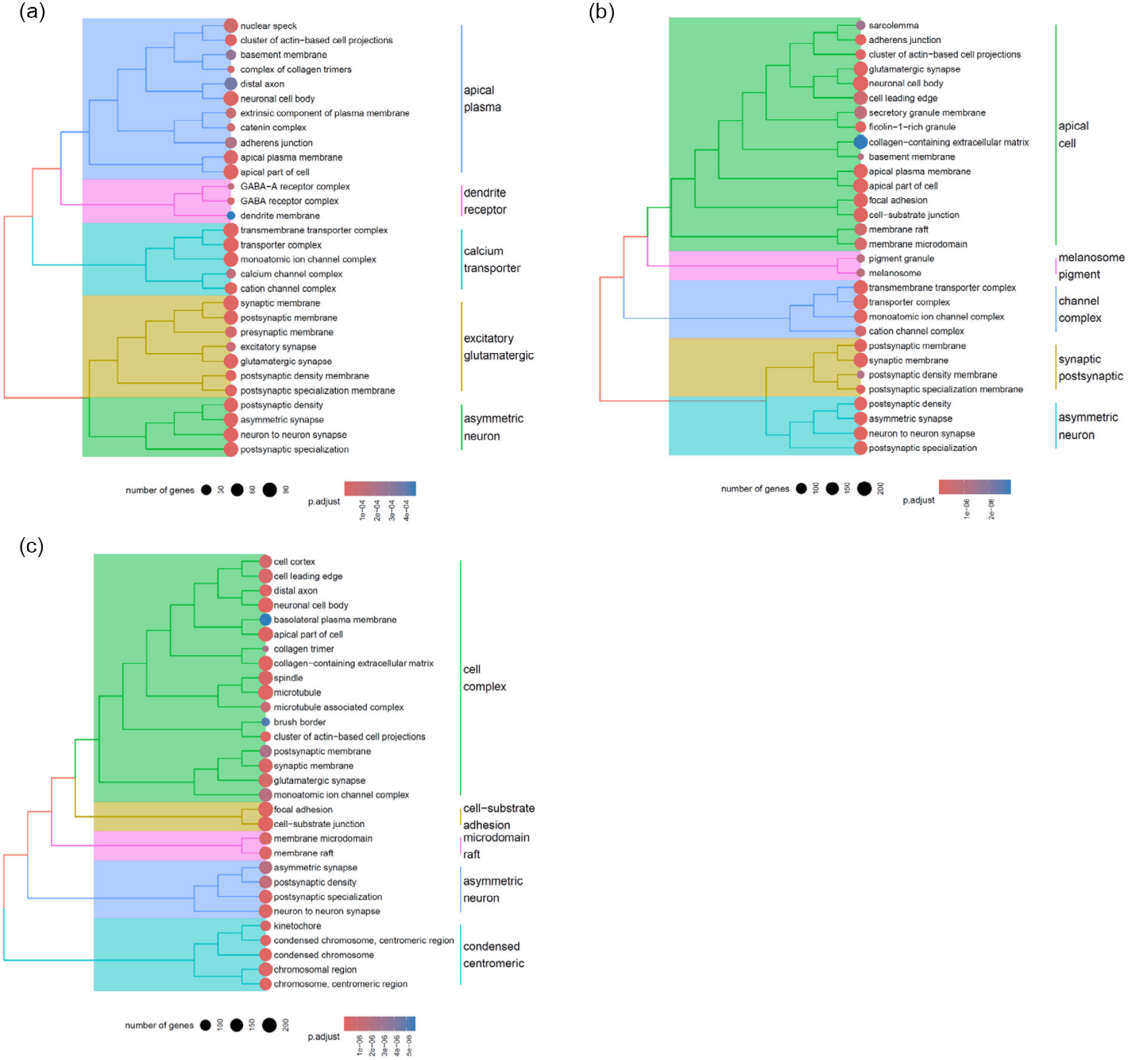
GO enrichment analysis of neuron‐like cells exposed to space‐related stressors. (a) Effect of µ*g* alone, (b) effect of CR alone, (c) combined effect of µ*g* and CR. CR = Cosmic radiation; GO = gene ontology.

**FIGURE 7:**
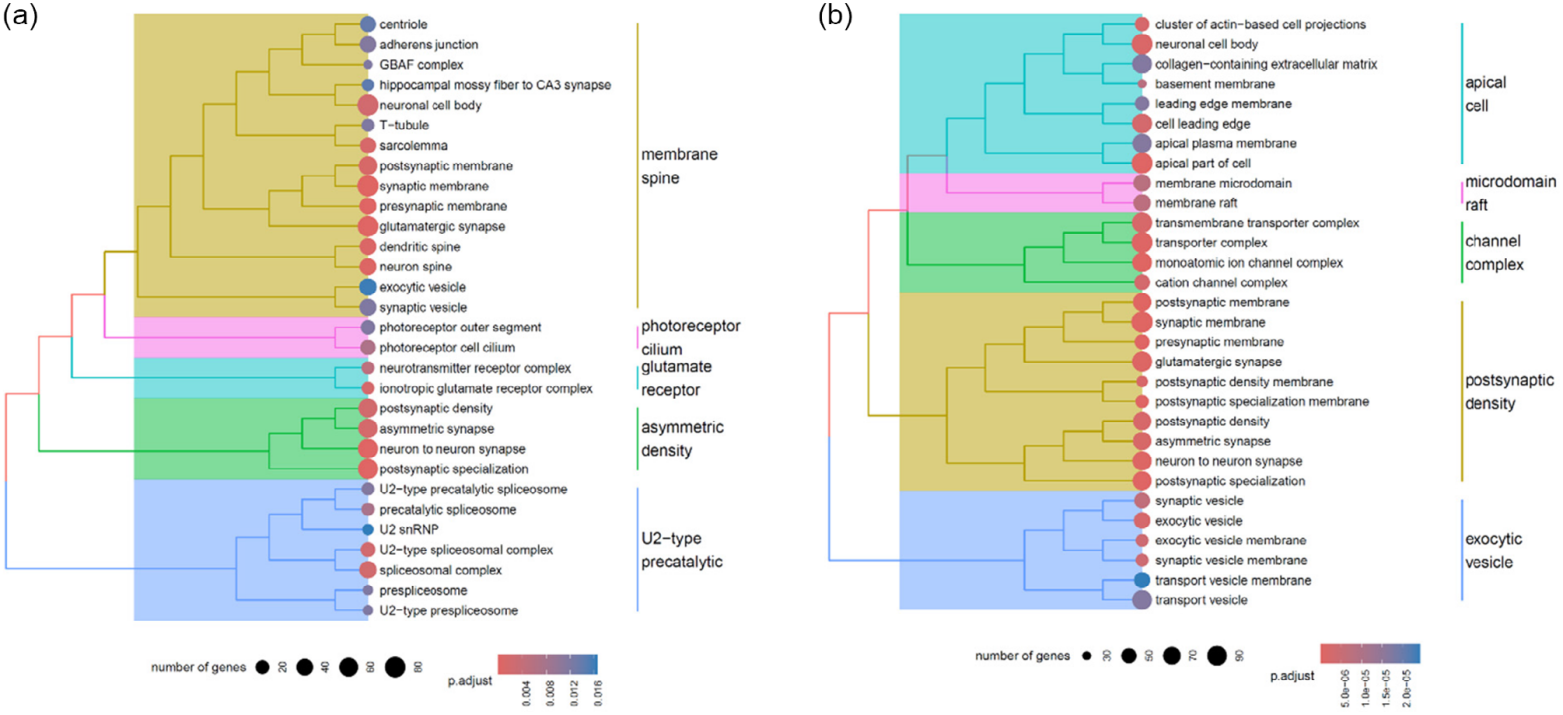
GO enrichment analysis of neuron‐like cells treated with NC. (a) Effect of nanoceria at 1*g*, (b) protective effect of nanoceria against combined µ*g* and CR. CR = Cosmic radiation; GO = gene ontology.

Volcano plots, clustered heatmaps, and gene ontology (GO) enrichment outputs for these comparisons are shown in Figures 4, 5, 6, and 7.

## 3 Discussion

To date, only a few studies have examined CNS‐adaptive responses to the combined space stressors of gravitational unloading and ionizing radiation. Among them, an animal study implying a 21‐day hindlimb unloading coupled with chronic low‐dose *γ‐*irradiation produced far broader long‐term brain transcriptomic and promoter‐methylation alterations in mice than either stressor alone, with enrichment in neurogenesis/neuroplasticity and neuronal signaling pathways after readaptation [23]. In a complementary behavioral study from Bellone et al., the combination of sµ*g* with chronic low‐dose *γ‐*irradiation was associated with blood–brain barrier dysfunction and changes in animal exploratory behavior that were not reproduced by single exposures [24]. However, stressor interactions are not universally synergistic. For example, Roggan et al. showed radiation‐quality‐dependent DNA damage responses in primary murine astrocytes, while clinostat‐obtained sµ*g* did not measurably alter DNA double‐strand break repair [25]. To address the existing gap in our understanding of how µ*g* and CR affect the nervous system, this work explored cerium oxide nanoparticles as a candidate neuroprotective strategy for neurons undergoing spaceflight conditions.

Consistent with our previous work [26], the synthesized nanoceria displayed an average core diameter of ≈13 nm, with SAED patterns and XPS spectra characteristic of crystalline cerium oxide [27]. XPS analysis, in particular, revealed that the Ce^4+^ oxidation state is predominant over the Ce^3+^ state, a redox profile compatible with catalase‐like antioxidant behavior of the nanostructures [28]. When coated with FBS, nanoceria formed a nearly monodisperse population with a mean *D*_h_ of about 200 nm and a strongly negative *ζ*‐potential. Nanoceria maintained long‐term colloidal stability in the differentiation medium used for neuron‐like cells, indicating suitability for the time frames associated with payload preparation and delivery to the ISS [29].

Then, we studied how nanoceria interacts with neuron‐like cells by first assessing their effects on cell viability. As noted above, nanoceria were highly biocompatible under both standard gravity and sµ*g*, with negligible toxicity across the tested conditions. In addition, nanoceria treatment significantly counteracted oxidative stress following a pro‐oxidant challenge, although the protective effect was partially attenuated in sµ*g* compared with 1 g. We also investigated the ability of nanoceria to foster neuronal differentiation. Nanoceria exposure led to a robust increase in differentiation indices, with an approximate twofold rise in median neurite length. A similar, enhancement was observed under sµ*g*, although of a smaller extent. By dampening oxidative stress, nanoceria appear to create a more permissive redox environment that favors neuronal maturation and neurite outgrowth [30]. Collectively, these observations support the potential of nanoceria as a tool for promoting neuronal regeneration.

To assess nanoceria neuroprotective capacity in the spaceflight setting, we carried out a detailed transcriptomic study. This approach allowed us to probe, at the molecular level, which cellular pathway might be modulated by nanoceria while cells were simultaneously challenged by µ*g* and CR. For the in‐flight samples, RNA‐seq reads aligned with *Homo sapiens* sequences, and in a few samples also with *Bacillus* spp. of unclear provenance, indicating accidental microbial contamination at payload integration. This issue was confined to two experimental conditions and primarily resulted in a reduced number of usable replicates, yet it did not preclude extraction of informative transcriptomic patterns. In the discussion below, we therefore interpret the results with a focus on pathways linked to oxidative stress control and neuroprotective mechanisms.

The analysis of the effects of µ*g* alone (comparison A *vs.* C) revealed a significant enrichment of numerous GO terms related to neuronal function, including *dendritic spine*, *postsynaptic membrane*, and *glutamatergic synapse*. This enrichment was found upon observation of the downregulation of a large set of genes with key roles in neuronal function. Among these downregulated markers, there is *NTRK3*, with a log_2_ fold change of −3.1, encoding for a member of the neurotrophic tyrosine receptor kinase (NTRK) family, precisely the receptor for neurotrophin‐3 (NT‐3), supporting neuronal differentiation and synapse development/plasticity [31]. Previous in vivo studies showed how NT‐3 deficiency is strongly associated with loss of specific sensory neuron populations and circuit deficits, indicating vulnerability when signaling is lowered [32]. *DCC* (log_2_ fold change −1.9) is another marker that underwent downregulation, which is associated with callosal dysgenesis and congenital mirror movements, consistent with altered neuronal wiring [33, 34]. This effect is attributable to the role of *DCC*, which encodes for netrin‐1 receptor, essential for axon guidance and is implicated in later circuit maturation (*e.g.*, dopamine pathway wiring) [35]. µ*g* exposure was also found to downregulate, with a log_2_ fold change of −3.1, *RBFOX1*, which encodes for an RNA‐binding protein that regulates tissue‐specific alternative splicing, crucial for neuronal development, synaptic genes, and circuit function [36]. CNS‐specific loss of *RBFOX1* disrupts neuronal splicing networks and is linked to altered neuronal physiology/brain function in genetic models, consistent with broad impacts on excitability [37]. Another marker that we found to be downregulated by the exposure to µ*g* is *GRIN2A*, with a log_2_ fold change of −4.1. Encoding for the GluN2A subunit of NMDA receptors, the expression of *GRIN2A* is crucial for excitatory synaptic transmission, calcium signaling, and forms of synaptic plasticity [38]. Reduction of GluN2A levels leads to dendritic structure/spine properties changes, potentially impacting neuronal circuit refinement and plasticity [39]. We also observed interference with the mitochondrial activity. Specifically, lower expression of *PRKN* (log_2_ fold change −1.1), which encodes for parkin, an E3 ubiquitin ligase that plays a central role in mitophagy [40]. Reduced parkin activity impairs mitophagy, promoting mitochondrial dysfunction and an increase of oxidative stress; also, *PRKN*‐deficient patient‐derived neuronal models show mitochondrial/ROS abnormalities consistent with this vulnerability [41].

Similarly, in the case of exposure to CR (comparison A *vs.* E), we observed the enrichment of multiple GO terms associated with synaptic activity, such as *postsynaptic density*, *cation channel complex*, and *neuron‐to‐neuron synapse*, again depending on the downregulation of several genes indicative of neuronal impairment. Among those genes, there is *SLC1A2* (log_2_ fold change of −2.4), encoding for an astrocytic glutamate transporter that prevents extracellular glutamate accumulation [42]. Its downregulation can lead to excitotoxicity, an excessive stimulation of neurons by excitatory neurotransmitters (in this case: glutamate), leading to an overload of calcium ions and causing neuronal damage and death through the activation of destructive enzymes, oxidative stress, and mitochondrial dysfunction [43]. Another downregulated marker is *OXR1* (log_2_ fold change −1.2), involved in response to oxidative stress by the production of the oxidation resistance protein 1, which supports neuronal survival under oxidative/DNA‐damage stress [44]. In case of downregulation, a weaker protection against ROS‐driven damage was observed, potentially increasing vulnerability to degeneration‐like phenotypes [45]. Similarly, *GRIN2B* (log_2_ fold change −4.1) encodes for the GluN2B subunit of NMDA receptors, crucial for excitatory synaptic transmission, calcium signaling, and synaptic plasticity [46]. Its downregulation alters NMDA receptor composition/signaling, thus impairing plasticity/cognition‐related pathways, along with alterations about how neurons handle Ca^2+^ loads under stress [47, 48]. Furthermore, our results suggest that CR may also affect the regulation of genes that encode for growth factors, such as *NGF* (log_2_ fold change −1.2), responsible for producing the nerve growth factor, which is essential for neuronal survival and differentiation [49]. Reduced production of neurotrophin leads to a reduction of trophic support and stress resilience in neurons, potentially worsening oxidative/inflammatory damage and contributing to functional deficits [50]. As well as µ*g*, downregulation due to CR was also observed in genes involved in mitochondrial function. Of these, *PPARGC1A*, which showed a log_2_ fold change of −2.0, encodes for a protein involved in the regulation of mitochondrial biogenesis and oxidative metabolism [51]. *PPARGC1A* downregulation leads to impaired mitochondrial capacity and redox buffering, therefore higher susceptibility to ROS, energetic stress, and synaptic dysfunction [52].

The evaluation of the synergistic effects of µ*g* and CR (comparison A *vs.* G) highlighted the enrichment of several GO terms identified in the previous comparisons, including *focal adhesion*, *synaptic membrane*, and *postsynaptic specialization*, resulting from the downregulation of genes already highlighted in the previous comparisons on the effects of µ*g* or CR alone. Of particular interest, we identified several genes that were consistently downregulated across all three conditions, namely when µ*g* or CR was considered individually, as well as when they acted in combination. An example, with a log_2_ fold change of −2.1, is provided by *CACNA1A*, encoding for a subunit of presynaptic calcium channel controlling Ca^2+^ entry that triggers neurotransmitter release, especially critical in cerebellar/neuronal circuits [53]. Studies have shown that reduced expression of this gene leads to weaker synaptic transmission and neurotransmitter release, and consequently to neurological dysfunction, such as ataxia, migraine, and epilepsy [54]. Across the three comparisons, we also found downregulated *MET* (log_2_ fold change −2.4), encoding for a protein member of the receptor tyrosine kinase family, implicated in neurite outgrowth, synaptogenesis, and neural circuit maturation [55]. Lower *MET* expression is generally associated with reduced synaptic maturation, potentially contributing to hypoconnectivity or maladaptive synaptic development under stress conditions [56]. Similarly, with a log_2_ fold change of −2.6, *ALDH1A1*, encoding for the aldehyde dehydrogenase 1A1, helps limiting the accumulation of dopamine‐derived toxic aldehydes [57]. A reduced aldehyde dehydrogenase activity promotes cell stress and vulnerability (including *α*‐synuclein–related toxicity), consistent with *ALDH1A1* being protective for nigrostriatal neuron subpopulations [58]. Another notable marker that we found to be downregulated is *FOXP2* (log_2_ fold change −1.8), encoding for a transcription factor essential for corticostriatal circuit development and function, linked to speech/language‐related circuitry and motor sequence learning [59]. As observed in animal models, a decreased production of this factor can lead to alterations in social/communication‐related behaviors [60].

Even acknowledging the constraints arising from microbial contamination in a subset of the in‐flight cell cultures, the overall results indicate that µ*g* and CR lead to cellular responses indicative of compromised synaptic processes and perturbed mitochondrial homeostasis, changes that could undermine neuronal stability and function in the spaceflight environment.

The antioxidant activity of nanoceria was first evaluated under normal gravity conditions in control cells not exposed to CR (comparison H *vs.* G). This comparative approach revealed an enrichment of additional GO terms associated with neuronal activity, such as *neurotransmitter receptor complex*, *glutamate receptor complex*, and *endocytic vesicle*. Nanoceria treatment induced the differential expression of a large set of genes, including several markers implicated in antioxidant activity and neuronal support. For instance, with a log_2_ fold change of 6.75, we observed the upregulation of *MT‐RNR2*, encoding for the protein humanin, a mitochondrial‐derived peptide with strong antiapoptotic and neuroprotective activity [61]. Higher humanin production is associated with improved cell survival, protection against Alzheimer's disease‐relevant insults, and oxidative/proteotoxic stress [62]. Another notable example is *IKZF2*, which showed a log_2_ fold change of 1.3, encoding for a transcription factor involved in T cell regulation, thus implicated in maintaining immune regulatory programs [63]. The upregulation of *IKZF2* is generally consistent with stronger immune regulation, which is typically beneficial for tissue homeostasis when no pathogen threat is present [64]. *KCNIP4*, which showed a log_2_ fold change of 1.4, encodes for a member of the family of voltage‐gated potassium channel‐interacting proteins (KCNIPs), managing channel trafficking and inactivation kinetics [65]. Higher *KCNIP4* expression can shape neuronal excitability, potentially improving firing precision and reducing the risk of hyperexcitability in basal conditions [66].

Transcriptomic profiling of cells treated with nanoceria and exposed to space environment indicated that these particles possess potential as a neuroprotective agent (comparison B *vs.* A). Enrichment was observed in GO terms, such as *synaptic vesicle*, *postsynaptic density*, and *cation channel complex*, supporting the involvement of nanoceria in the modulation of pathways relevant to neuronal structure and function. Many of the genes that had been reported as downregulated by spaceflight‐related stressors were no longer downregulated once cells were treated with nanoceria. For example, genes associated with neuronal differentiation, such as *RBFOX1* and *MET*, as well as genes associated with neuronal plasticity, such as *GRIN2A* or *GRIN2B*, recovered their expression levels. Moreover, genes associated with mitochondrial activity, such as *PPARGC1A* and *PRKN*, and genes associated with dopamine metabolism, such as *ALDH1A1*, recovered their expression levels as well. In addition, several genes that had undergone downregulated expression were no longer suppressed after nanoceria administration. Another noteworthy example of a gene that recovered its normal expression level, thanks to treatment with nanoceria, is *GAL*, which encodes for galanin, a neuropeptide that modulates neuronal excitability and has neuroprotective effects in multiple injury or stress contexts [67]. In an animal model, galanin underproduction was associated with lower protection against oxidative/excitotoxic insults and worsened neuronal vulnerability under stress [68]. Also noteworthy is the *SPATA12* gene, implicated in cellular stress responses, including growth control and DNA damage‐related pathways [69]. In experimental models, lower *SPATA12* expression led to weakened cellular defense/repair programs (particularly relevant when radiation can induce DNA damage), potentially increasing stress sensitivity [70]. Similarly, *CNTNAP5*, encoding for a neurexin‐family cell‐adhesion molecule, recovered a normal expression level. Decreased *CNTNAP5* expression is detrimental by reducing synaptic adhesion/organization and neural network stability [71].

It should be emphasized that nanoceria did not completely revert to control values the expression levels of those genes that were altered by spaceflight‐related stressors. In several instances, the decrease in expression was reduced but not entirely reversed. For example, *HTR4* shifted from log_2_ fold changes of −2.3 to −1.0. This gene encodes for the serotonin 4 receptor, a G‐protein‐coupled receptor that stimulates cAMP production in response to serotonin, modulating the release of various neurotransmitters [72]. Similarly, *GFRA1*, encoding for GFRα1, involved in supporting neuronal survival and differentiation (notably dopaminergic neurons), shifted from log_2_ fold change of −1.3 to −1.1 [73, 74]. Partially recovered was also the transcription of *UNC5D* (log_2_ fold change from −1.4 to −1.0), encoding for a netrin receptor, thus involved in neuronal guidance and trophic factor‐linked survival programs [75].

Taken together, these results point to a strong neuroprotective effect of nanoceria. The treatment not only prevented the spaceflight‐associated downregulation of the transcription of multiple genes linked to neuronal communication and mitochondrial energy regulation but also helped upregulating the expression of additional targets that were only partly recovered. In doing so, nanoceria appear to support core pathways required for neuronal viability and performance under harsh spaceflight stressors.

In addition to evaluating the direct impact of nanoceria exposure, we examined the “rescue” scenario by contrasting stress‐exposed nanoceria‐treated cells with unstressed untreated controls. Notably, this rescue comparison yielded fewer significantly enriched GO categories than the other comparisons. The narrower set of enriched terms likely reflects a normalization effect, suggesting that nanoceria helped shift cellular programs back toward a baseline, more physiological state. In particular, when examining rescue under µ*g* (comparison B *vs.* C), we found that roughly 92% of the genes significantly downregulated by µ*g* alone were no longer downregulated in cultures supplemented with nanoceria. This indicates an almost complete rescue of neuronal damage by nanoceria in µ*g* conditions. In the combined µ*g* and CR scenario (comparison B *vs.* G), we observed that about 93% of the transcripts that were significantly suppressed by the combination of µ*g* and CR were no longer downregulated when cells were treated with nanoceria. This pattern underscores once more the nanoparticle beneficial effects toward neurons.

Both nanoceria and PDNPs were investigated as antioxidant countermeasures against µ*g* and CR‐induced neuronal damage using the same experimental platform [17]. Despite their markedly different physicochemical nature, NC being inorganic redox‐active nanozymes and PDNPs being organic melanin‐mimetic nanostructures, both systems converged on a robust neuroprotective outcome. At the cellular and functional level, both nanoparticles demonstrated excellent biocompatibility in neuron‐like SH‐SY5Y cells and significantly mitigated oxidative stress. PDNPs reduced cytosolic and mitochondrial ROS while preserving intracellular GSH levels under both 1 g and sµ*g*, even following pro‐oxidant challenge. Similarly, nanoceria preserved GSH content and counteracted ROS accumulation, with a slightly attenuated but still significant effect under sµ*g* conditions. In both cases, reduced efficacy in µ*g* was attributed to altered nanoparticle–cell interactions rather than intrinsic loss of antioxidant activity. Both nanomaterials also promoted neuronal differentiation, as evidenced by increased neurite length and neurite number. PDNPs approximately doubled neurite extension under 1 g and significantly enhanced differentiation under sµ*g*, consistent with their catechol‐rich, dopamine‐like chemistry. Nanoceria induced a comparable enhancement of neurite outgrowth, likely through sustained redox buffering and mitochondrial protection. These findings indicate that restoration of a permissive redox environment is sufficient to support neuronal structural plasticity, regardless of nanoparticle composition.

At the transcriptomic level, the two nanoparticles showed striking parallels. Spaceflight exposure (µ*g* and/or CR) induced widespread downregulation of genes involved in synaptic organization, mitochondrial metabolism, and dopaminergic signaling. Treatment with either PDNPs or nanoceria substantially reversed these alterations. In PDNP‐treated cells, key pathways related to antioxidant defense, mitochondrial integrity, and dopamine metabolism were stabilized, with near‐complete rescue of µ*g*‐induced transcriptional damage and partial rescue of CR‐driven alterations. Nanoceria produced a similarly strong normalization of gene expression, restoring the majority of gravity‐ and radiation‐sensitive transcripts toward ground‐control levels. Notably, both treatments preserved the expression of genes central to synaptic transmission, energy metabolism, and dopamine handling, underscoring convergent molecular targets.

Mechanistically, key differences emerge. Nanoceria act through regenerative Ce^3+^/Ce^4+^ redox cycling, conferring prolonged, enzyme‐like ROS scavenging capacity. PDNPs, instead, rely on multifunctional catechol chemistry that combines radical scavenging with neuromelanin‐ and dopamine‐mimetic properties, potentially influencing dopamine stability and intracellular signaling more directly. Despite these differences, both materials ultimately buffer oxidative stress, which appears to be the dominant upstream driver of the observed neurodegenerative signatures.

Summarizing, the two studies demonstrate that distinct nanoantioxidant platforms can achieve overlapping neuroprotective outcomes in spaceflight conditions. Both nanoceria and PDNPs preserve neuronal redox homeostasis, support neurite outgrowth, and normalize transcriptomic programs disrupted by µ*g* and CR. These findings highlight oxidative stress as a central therapeutic target in space‐induced neurodegeneration and suggest that multiple nanotechnological strategies, organic or inorganic, can be successfully deployed to protect CNS function during long‐duration missions and, potentially, in oxidative stress‐related neurodegenerative diseases on Earth.

## 4 Conclusions

Microgravity and CR experienced during spaceflight place neuronal cells under pronounced oxidative pressure, with downstream signatures consistent with synaptic dysfunction, impaired mitochondrial energy homeostasis, and alterations in dopamine‐related pathways. Together, these molecular shifts provide a plausible mechanistic basis for the motor and cognitive vulnerabilities reported by astronauts during long‐duration missions. In this study, we show that cerium oxide nanoparticles, exploited for their redox cycling capacity, substantially attenuate space‐relevant oxidative damage and reshape the neuronal transcriptomic response both in flight and in ground‐based conditions. Under combined exposure to microgravity and CR, nanoceria restore the expression of most gravity‐sensitive genes toward baseline, and significantly attenuate the magnitude of radiation‐associated perturbations. These findings support the idea that nanoceria buffer convergent stress pathways triggered by gravitational unloading and ionizing radiation, helping preserve molecular programs required for neuronal function. Beyond the spaceflight context, our results highlight nanoceria as a versatile platform to rapidly interrogate antioxidant‐based interventions for neurological conditions where oxidative stress and mitochondrial dysfunction are central drivers, including Parkinson's disease and related neurodegenerative disorders. Future work will focus on validating these protective effects in more complex biological systems, expanding testing to additional space‐relevant mission profiles, and advancing translational evaluation on Earth. Ultimately, integrating nanoceria with established countermeasures, such as exercise, targeted nutrition, and selected pharmacological approaches, may enable multilayered strategies to safeguard brain health in extreme environments and across aging populations.

## 5 Experimental Section

### 5.1 Nanoparticle Synthesis and Characterization

Nanoceria were synthesized following a chemical precipitation method described by Genchi et al. [26]. In brief, cerium (III) nitrate was used as a precursor in aqueous solution and reacted with a base (*i.e.*, ammonium hydroxide) under stirring, leading to the formation of cerium oxide nanocrystals. The plain nanoceria were extensively washed by repeated centrifugation and resuspension in deionized water to remove residual reagents. Thereafter, nanoparticle stability in aqueous medium was obtained by coating with FBS. Specifically, nanoparticles were dispersed at 2 mg/mL in sterile 40% (*v*/*v*) FBS and incubated for 1 h at room temperature under gentle agitation. This incubation allows a protein corona to adsorb onto the nanoceria surface, yielding FBS‐coated nanoceria. The nanoparticle dispersion in FBS was diluted 1:20 in the culture medium to obtain a final concentration of 100 µg/mL.

FBS‐uncoated nanoparticle morphology and size were characterized by TEM imaging. A drop of 50 µg/mL nanoceria suspension was deposited on a carbon‐coated copper grid and then imaged with a JEM‐1400Plus system (JEOL) equipped with a LaB_6_ thermionic source operating at 120 kV. Average diameter was determined from acquisitions by measuring at least 100 nanoparticles with Gwyddion software.

Electron diffraction was performed with a JEM‐1400Plus system (JEOL) equipped with a LaB_6_ thermionic source operating at 120 kV in SAED mode to observe the crystalline nature of the cerium oxide nanoparticles.

XPS analysis was performed on uncoated nanoparticles deposited onto an indium pellet with a Kratos Axis Ultra DLD spectrometer, equipped with a monochromatic Al Kα source operating at 15 kV and 20 mA. A wide‐scan spectrum was acquired with 160 eV pass energy, while a high‐resolution narrow‐scan spectrum was obtained with a constant 10 eV pass energy and steps of 0.1 eV. Photoelectrons were detected at a take‐off angle *φ* = 0° with respect to the surface. The charging shift was calibrated with the binding energy of the C 1s as a baseline (284.8 eV). Data were acquired at a pressure lower than 7·10^–9^ Torr in the analysis chamber, and they were converted to VAMAS format and processed by using the CasaXPS 2.3.22 software.

*D*_h_ and *ζ*‐potential of FBS‐coated nanoceria were analyzed by DLS measurements, by using a Malvern Zetasizer Nano ZS90. Nanoceria dispersion at 100 µg/mL was loaded into disposable cuvettes to determine *D*_h_ and PDI, or into capillary cells for *ζ*‐potential analysis. All measurements were performed at 25°C. Additionally, a nanoparticle colloidal stability test was conducted to ensure that the FBS‐coated nanoparticles remained stable in the cell culture conditions over the experimental timeframe. Nanoceria at a concentration of 100 µg/mL were incubated at 37°C in differentiation medium composed of high‐glucose Dulbecco's modified Eagle's medium/F‐12 with 15 mM HEPES (DMEM/F12, Gibco), supplemented with 2% FBS (Gibco), 1% *L*‐glutamine (200 mM stock, Gibco), 10 µM retinoic acid (RA, Thermo Scientific), and 1% penicillin–streptomycin (100 IU/mL penicillin, 100 µg/mL streptomycin, Gibco). *D*_h_ and PDI values were recorded every 10 min for 1 h, and then samples were kept at 37°C and analyzed every 48 h for 10 days.

### 5.2 Cell Culture

Human SH‐SY5Y neuroblastoma cells (HTL95013, ICLC) were used as a neuron‐like cell model. Cells were grown in DMEM/F‐12 supplemented with 10% FBS, 1% *L*‐glutamine, and 1% penicillin–streptomycin, and maintained at 37°C in a humidified 5% CO_2_. The proliferation medium was replaced with the low serum medium described in the previous paragraph for differentiation induction.

### 5.3 Nanoparticle–Cell Interaction

In vitro studies on nanoparticle–cell interactions were carried out by using differentiated SH‐SY5Y neuroblastoma cells maintained either at 1 g or under sµ*g*. Simulated gravity conditions were obtained with a random positioning machine (RPM, Airbus 2.0) operated at an angular speed of 25°–60°/s. The “home” position of the instrument was defined as the orientation in which the sample holder was perpendicular to the 1 g gravity vector, while the inner and outer rotational frames were, respectively, arranged parallel and orthogonal to this vector.

Cell viability in response to nanoceria exposure was quantified by using the Quant‐iT PicoGreen dsDNA Assay Kit (Invitrogen). SH‐SY5Y cells were seeded in 96‐well plates (Corning) at a density of 10,000 cells/cm^2^ and maintained in proliferation medium for 24 h. Subsequently, cells were treated with increasing concentrations of nanoceria (0–200 µg/mL) in differentiation medium for either 48 or 96 h. At the end of the exposure period, cultures were rinsed with DPBS and lysed in 100 µL of ultrapure water by subjecting them to three freeze–thaw cycles between −80°C and 37°C. The assay was then carried out by combining the cell lysates with PicoGreen reagent and Tris–EDTA buffer in black 96‐well polystyrene plates (Corning Costar), in accordance with the manufacturer's protocol. Fluorescence was recorded at 485 nm excitation and 535 nm emission by using a Victor X3 Multilabel Plate Reader (Perkin Elmer).

To assess the antioxidant properties of nanoceria in neuron‐like cells, we used a GSH Assay Kit (Abcam). This assay is based on the glutathione recycling system to produce a yellow chromophore that enables the quantification of GSH levels by monitoring absorbance. Cells were plated in 24‐well plates at a density of 10,000 cells/cm^2^ and maintained for 24 h in proliferation medium. They were then exposed for 96 h to 100 µg/mL nanoceria, 30 nM TBH as pro‐oxidant stimulus, or a combination of both, yielding four conditions: Control, NC‐treated, TBH‐treated, and NC + TBH cotreated. At the end of the incubation, cells were collected and processed following the manufacturer's instructions. Briefly, once detached, cells were lysed on ice, centrifuged, and the GSH‐containing supernatant was collected and reacted with the assay mixture for quantification. Absorbance was subsequently read at 415 nm by using a Victor X3 Multilabel Plate Reader (Perkin Elmer). Results were normalized to the number of cells used for each experimental condition.

To examine whether nanoceria influences neuronal differentiation, SH‐SY5Y cells were plated at low density (1,000 cells/cm^2^) in CELLview dishes. After an initial 24 h period in proliferation medium, cultures were maintained for 96 h in differentiation medium, either in the absence or presence of 100 µg/mL nanoceria. Cells were then fixed in 4% paraformaldehyde in DPBS for 20 min at 4°C and immunolabeled for tubulin β‐III. The staining procedure involved permeabilization with 0.1% Triton‐X in DPBS for 15 min, blocking with 10% goat serum (GS) in DPBS for 1 h, and incubation for 3 h with a rabbit antitubulin β‐III antibody (0.3 µg/mL, Sigma) diluted in 10% GS. After four rinses with 10% GS for 5 min, samples were incubated with a 10% GS solution containing a goat antirabbit Alexa Fluor 488‐conjugated secondary antibody (10 µg/mL) and Hoechst 33 342 (5 µg/mL) for 40 min. After three washes in DPBS, samples were imaged by using a Nikon Eclipse Ti epifluorescence microscope equipped with a 10× objective, and quantitative image analysis was carried out with ImageJ software.

### 5.4 In‐Flight Experiment

Neuron‐like cells were plated at 1,000 cells/cm^2^ on Thermanox coverslips (10.5 × 22.0 mm, Nunc) positioned in custom‐made silicone multiwell plates at approximately L − 96 h. An outline of the experimental schedule is shown in Figure S6a. At L − 77 h, the coverslips were transferred into 12 EUs (KEU‐ST, developed by Kayser Italia and flight‐qualified for use on the ISS, providing the first containment level). Each EU featured five reservoirs (1.3 mL each), which were filled as follows. Reservoirs 1 and 2 contained HEPES‐buffered differentiation medium, either without or with 100 µg/mL nanoceria; reservoirs 3 and 4 were loaded with DPBS for rinsing cells before fixation; reservoir 5 was filled with RNAlater (Ambion AM7020) to fix and preserve nucleic acids. A schematic of the EU fluidic architecture is shown in Figure S6b. For each condition, EUs (*n* = 3 per group) were placed inside an experimental container (EC; KIC‐SL, developed by Kayser Italia and flight‐qualified for use on the ISS), which supplied the second containment level and allowed interface with the European Space Agency (ESA) Kubik incubator aboard the ISS. Selected ECs carried iButton devices for temperature logging. From payload handover through its integration into the Kubik incubator, all samples were maintained at 37°C under active thermal control. The payload was launched on a Crew Dragon Freedom/Falcon 9 spacecraft (SpaceX). Within 24 h of docking to the ISS, ECs were inserted into the Kubik incubator preset to 37°C (Figure S7), and the first of five automated fluidic operations was initiated. Differentiation medium (with or without nanoceria) from reservoirs 1 and 2 was released at L + 63 h and L + 111 h, respectively. DPBS from reservoirs 3 and 4 was dispensed at L + 159 h with a 2 min interval between the two releases, followed 2 min later by the activation of reservoir 5 (RNAlater fixative). Two h after fixation, ECs were moved to the −80°C Minus Eighty‐Degree Laboratory Freezer for ISS and stored at −80°C until their return to Earth. An identical experimental protocol was reproduced on the ground by using an RPM to obtain sµ*g*.

### 5.5 Transcriptomic Analyses

Exposure to µ*g* and CR in the presence or absence of nanoceria generated eight distinct experimental conditions: A (‐NC, µ*g*, +CR), B (+NC, µ*g*, +CR), C (‐NC, s1*g*, +CR), D (+NC, s1*g*, +CR), E (‐NC, sµ*g*, ‐CR), F (+NC, sµ*g*, ‐CR), G (‐NC, 1*g*, ‐CR), H (+NC, 1*g*, ‐CR). From the full set of possible pairwise contrasts, seven key comparisons were selected (in each pair, the second group serves as the control): A *vs.* C to assess the specific contribution of µ*g*; A *vs.* E to discriminate the effect of CR only; A *vs.* G to quantify the overall impact of spaceflight‐associated stressors; H *vs.* G to determine the intrinsic effect of nanoceria alone; B *vs.* A to examine the neuroprotective action of nanoceria in true spaceflight conditions; B *vs.* C to assess nanoceria‐mediated rescue from µ*g*; B *vs.* G to evaluate the rescue provided by nanoceria under spaceflight conditions.

After hardware deintegration for both the in‐flight and ground‐based experiments, cell cultures were first inspected by phase‐contrast microscopy (Nikon Eclipse Ti), and temperature profiles from data loggers were analyzed to confirm that environmental conditions had remained compatible with cell survival. Cells were then transferred into 2 mL tubes, covered with their corresponding supernatants (containing > 70% RNAlater), and centrifuged at 16,000 g for 15 min at 4°C to collect cell pellets. Total RNA was isolated and purified by using the mirVana PARIS Kit (Ambion AM1556), following the supplier's recommendations. RNA quality and quantity was assessed with a NanoDrop 2000 spectrophotometer (Thermo Scientific).

A quantity of 300 ng of total RNA underwent rRNA depletion and RNA library building with NEBNext rRNA Depletion Kit (Human/Mouse/Rat) | NEB and NEBNext Ultra II RNA Library Prep Kit for Illumina | NEB kits. Final libraries were quantified by using a Qubit DNA assay (Thermo Fisher Scientific) and NanoDrop, and their fragment‐size distribution was evaluated on an Agilent 2100 Bioanalyzer. Quantitative real‐time PCR was then used to determine the number of amplifiable sequencing templates. Sequencing was carried out on an Illumina NovaSeq 6000 platform in paired‐end mode, and raw output reads were converted into FASTQ files for downstream analysis.

### 5.6 Bioinformatic Processing

FASTQ files first underwent quality control using FastQC (v.0.12.0). High‐quality reads from the standard libraries were then aligned to the human reference genome (hg38, Gencode annotation) [76] with STAR v2.7.11b [77], producing BAM alignment files. Gene‐level quantification was obtained with featureCounts [78] (Subread v2.18.0) [79], restricting the analysis to uniquely mapped reads falling within annotated exons. Differential expression analysis was performed using DESeq2 v1.44.0 [80]. Transcripts were classified as differentially expressed when they satisfied both the following thresholds: absolute log_2_ fold change (FC) > 1 and adjusted *p*‐value (Wald test) < 0.05. This pipeline generated seven distinct DEG sets, which were visualized by heatmaps (pheatmap v1.0.12) and Volcano plots (EnhancedVolcano v1.22.0) [81]. To elucidate the functional implications of each condition, GO enrichment was carried out with clusterProfiler v4.12.0 [82], applying a significance cutoff of 0.05 for both *p*‐ and *q*‐values.

### 5.7 Statistical Analysis

All statistical analyses were performed in *R*. Data distribution was first examined with the Shapiro–Wilk test. When normality was confirmed, group differences were evaluated by using ANOVA, followed by LSD *post hoc* tests with Bonferroni's adjustment, and values were reported as mean ± standard deviation. For datasets that did not meet normality assumptions, the Kruskal–Wallis test was applied, followed by Wilcoxon *post hoc* comparisons with Holm's correction; in this case, results were reported as median ±95% confidence interval. Unless otherwise specified, all experiments were conducted in triplicate (*n* = 3). For transcriptomic statistical analyses, please refer to the specific experimental section.

## Supporting Information

Additional supporting information can be found online in the Supporting Information section. NC DLS characterization; NC stability assessments; NC biocompatibility assessments; oxidative stress level analysis; thermal profile of the in‐flight experiment; timeline of the experiment and EU setup; EU assembly and placement in the incubator; DEG summary. **Supporting Fig. S1**: FBS‐coated nanoceria characterization. a) *D*_h_ distribution and b) *ζ*‐potential analysis. **Supporting Fig. S2**: Nanoceria stability assay performed in differentiation medium. Analysis of a, c) *D*_h_ and b, d) PDI values over a, b) 1 h and c, d) 10 days. **Supporting Fig. S3**: Biocompatibility assessments. a) PicoGreen assay, indicative of cell number, in a) 1g and b) sµ*g* (**p < 0.01). **Supporting Fig. S4**: Oxidative stress level analysis. Quantification of reduced glutathione (GSH) levels in a) 1g and b) µ*g* conditions (*n* = 3, ** *p* < 0.01, *** *p* < 0.001). **Supporting Fig. S5**: Thermal profile of the in‐flight experiment. The arrow indicates payload launch time. **Supporting Fig. S6**: In‐flight experiment. a) Experiment timeline with the indication of relevant time points (L = payload launch); b) diagram of the experimental flow circuit, depicting the positions of the five reservoirs: 1,2 differentiation medium; 3,4 DPBS; 5 fixative (CCC = cell culture chamber). **Supporting Fig. S7**; EUs integration. a–c) Representative pictures of the EUs integration procedure. d) EUs positioning inside the Kubik incubator/centrifuge. **Supporting Table 1**: Summary of differential expression analysis for protein‐coding genes.

## Author Contributions

**Alessio Carmignani**: iInvestigation; methodology; data curation; formal analysis; writing – original draft. **Attilio Marino**: methodology; data curation. **Matteo Battaglini**: methodology; data curation**. Nicoletta Di Leo**: investigation; methodology. **Elisa Carrubba**: investigation; methodology. **Michele Balsamo**: investigation; methodology**. Giovanni Valentini**: resources; supervision. **Gabriele Mascetti**: resources; supervision. **Serena Perilli**: resources; supervision. **Francesco De Boni**: methodology; data curation**. Sergio Marras**: methodology; data curation**.** **Mirko Prato**: methodology; data curation. **Giada Graziana Genchi**: conceptualization; resources; supervision; project administration; writing – review and editing. **Gianni Ciofani**: conceptualization; resources; supervision; project administration; writing – review and editing.

## Funding

This study was supported by Agenzia Spaziale Italiana (2021‐2‐R.1‐2023).

## Conflicts of Interest

The authors declare no conflicts of interest.

## Supporting information

Supplementary Material

## Data Availability

Omics data were deposited in NCBI Gene Expression Omnibus database and can be accessed through the GEO Series accession number GSE295652. All other data are available upon reasonable request to the Authors.
