# Supplementary material for: Neuroprotective Effects of Cerium Oxide Nanoparticles During Spaceflight
Source: Small Sci. 2026 Mar 31;6(4):e70271. doi: 10.1002/smsc.70271 (PMC13154917; doi:10.1002/smsc.70271)
Supplement: Supplementary file 1 — Supplementary Material [file SMSC-6-e70271-s001.pdf]

Neuroprotective Effects of Cerium Oxide Nanoparticles during Spaceflight

Alessio Carmignani[a,], Attilio Marino[a], Matteo Battaglini[a], Nicoletta Di Leo[a], Elisa Carrubba[b], Michele Balsamo[b], Giovanni Valentini[c], Gabriele Mascetti[c], Serena Perilli[c], Francesco De Boni[d], Sergio Marras[d], Mirko Prato[d], Giada Graziana Genchi[a,e,*,#], Gianni Ciofani[a,*,#]*

[a]Istituto Italiano di Tecnologia, Smart Bio-Interfaces, Viale Rinaldo Piaggio 34, 56025 Pontedera, Italy

[b]Kayser Italia S.r.l., Via di Popogna 501, 57128 Livorno, Italy

[c]Agenzia Spaziale Italiana, Via del Politecnico snc, 00133 Roma, Italy

[d]Istituto Italiano di Tecnologia, Materials Characterization Facility, Via Morego 30, 16163 Genova, Italy

[e]University of Bari "Aldo Moro", Department of Bioscience, Biotechnology and Environment, Via Orabona 4, 70125 Bari, Italy

*Corresponding Authors: alessio.carmignani@iit.it; giada.genchi@iit.it; gianni.ciofani@iit.it

[#]Equally contributing Authors

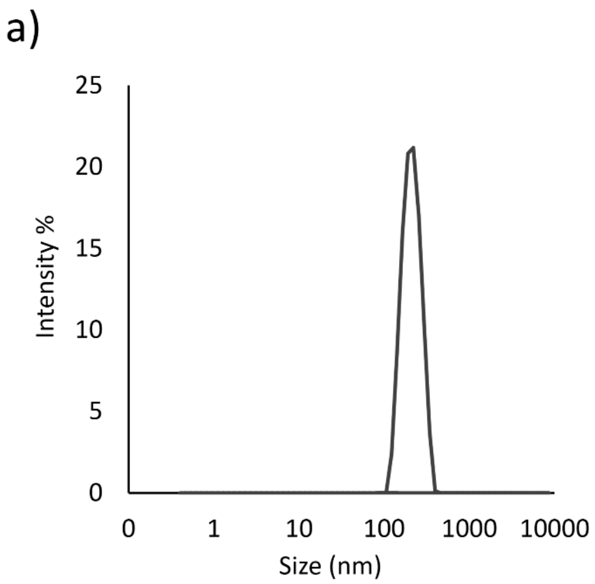

Figure S1. FBS-coated nanoceria characterization. a) D_h distribution and b) ζ-potential analysis.

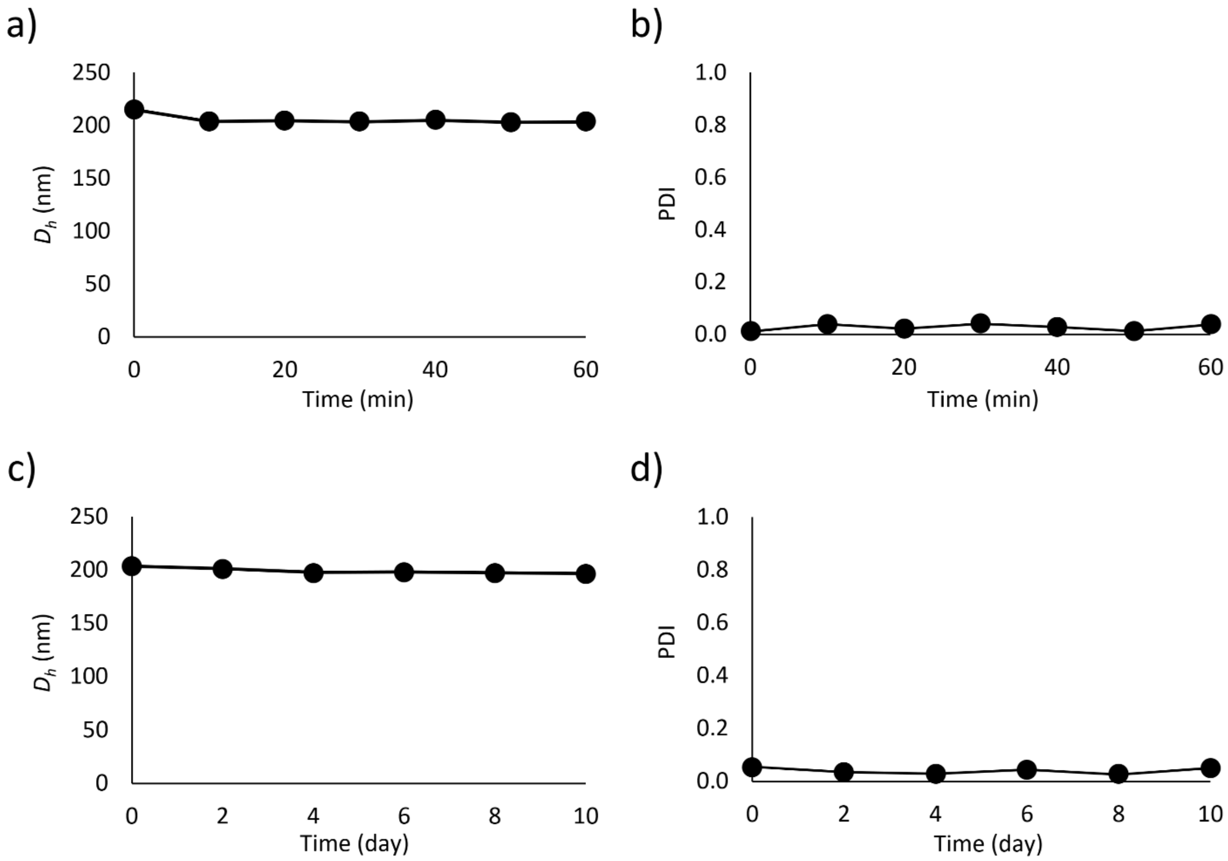

Figure S2. Nanoceria stability assay performed in differentiation medium. Analysis of a, c) D_h and b, d) PDI values over a, b) 1 h and c, d) 10 days.

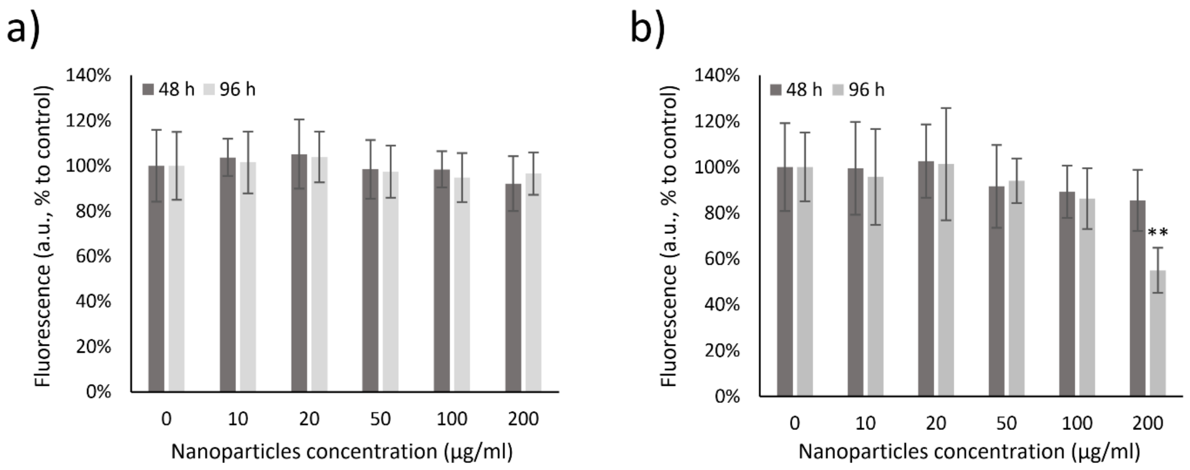

Figure S3. Biocompatibility assessments. a) PicoGreen assay, indicative of cell number, in a) 1*g* and b) sµ*g* (***p* < 0.01).

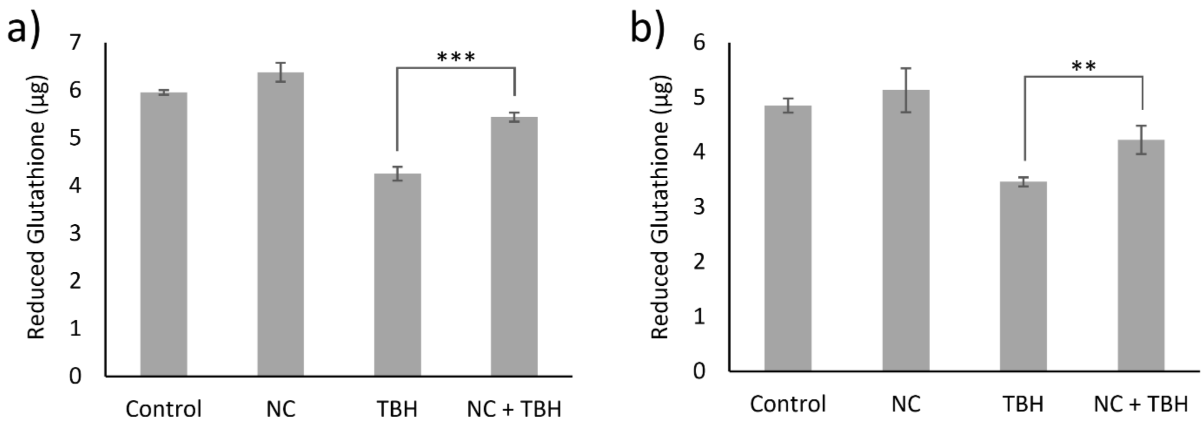

Figure S4. Oxidative stress level analysis. Quantification of reduced glutathione (GSH) levels in a) 1*g* and b) µ*g* conditions ($n = 3$, ** $p < 0.01$, *** $p < 0.001$).

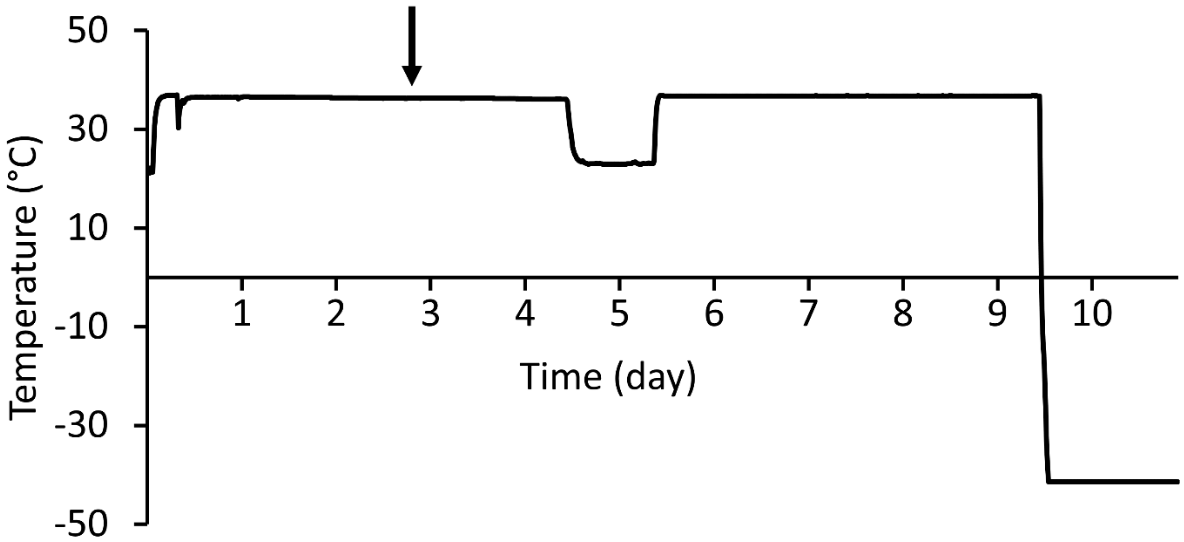

Figure S5. Thermal profile of the in-flight experiment. The arrow indicates payload launch time.

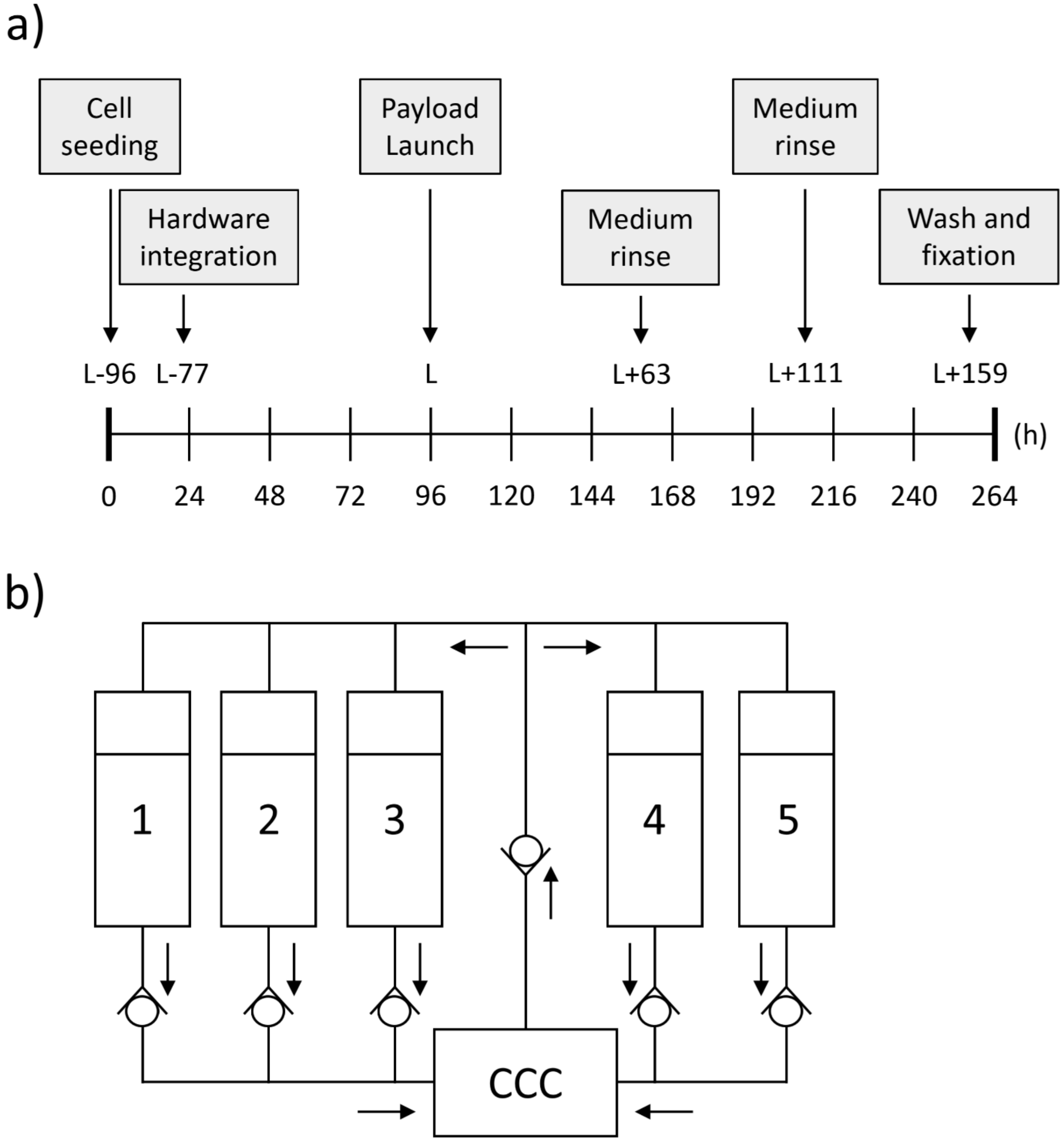

Figure S6. In-flight experiment. a) Experiment timeline with the indication of relevant time points (L = payload launch); b) diagram of the experimental flow circuit, depicting the positions of the five reservoirs: 1,2 differentiation medium; 3,4 DPBS; 5 fixative (CCC = cell culture chamber).

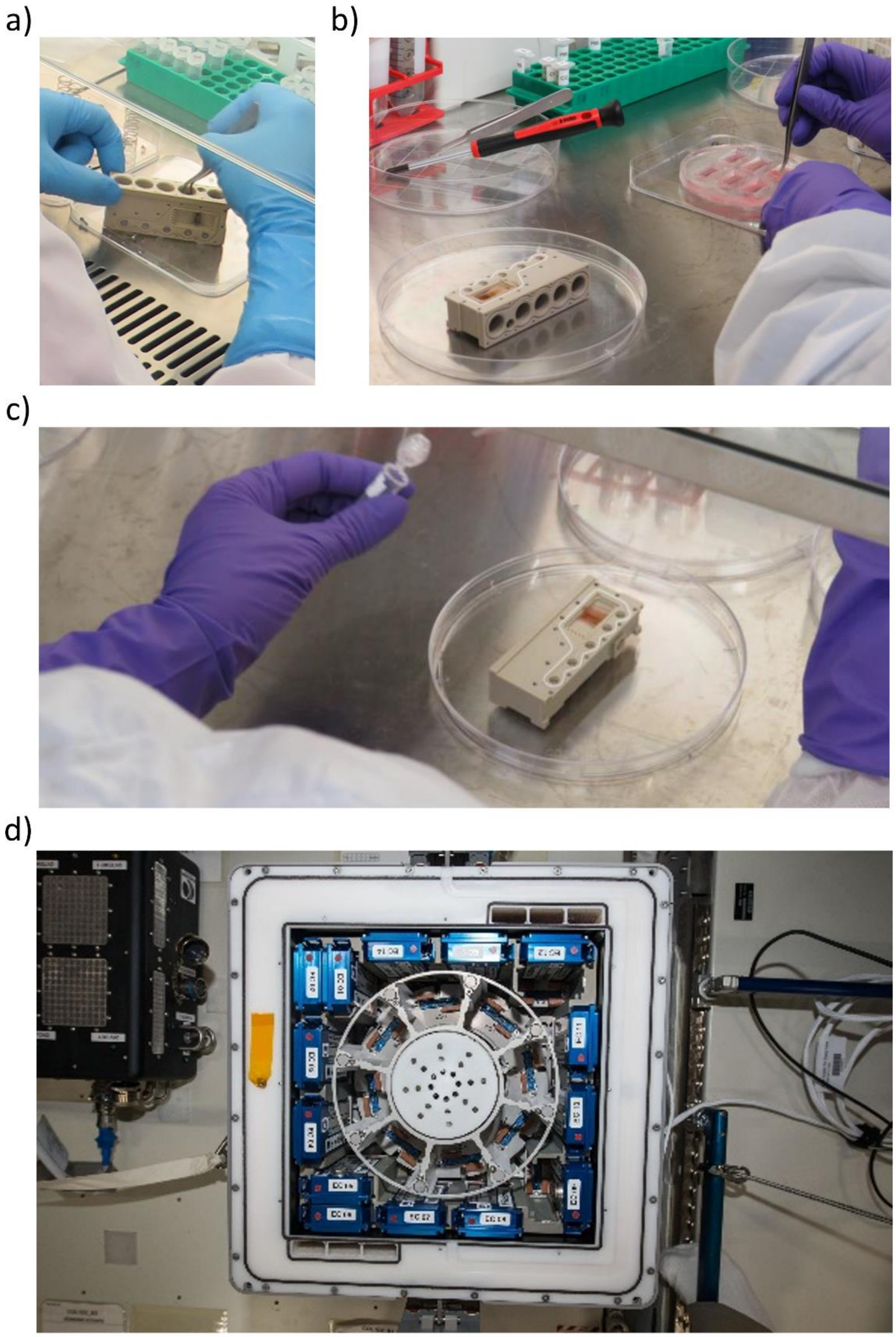

Figure S7. Experimental units (EUs) integration. a-c) Representative pictures of the EUs integration procedure. d) EUs positioning inside the Kubik incubator/centrifuge.

Table S1. Summary of differential expression analysis for protein-coding genes

Comparison	Upregulated genes	Downregulated genes	Total DEGs
A vs. C	42	936	978
A vs. E	409	3083	3492
A vs. G	530	2554	3084
H vs. G	61	6	67
B vs. A	800	52	852
B vs. C	127	237	364
B vs. G	322	155	477

Experimental classes: A (-NC, μ*g*, +CR); B (+NC, μ*g*, +CR); C (-NC, s1*g*, +CR); D (+NC, s1*g*, +CR); E (-NC, sμ*g*, -CR); F (+NC, sμ*g*, -CR); G (-NC, 1*g*, -CR); H (+NC, 1*g*, -CR).
